# Supplementary material for: Clinical and economic burden of varicella in pediatric patients hospitalized in four institutions in Guatemala
Source: PLOS Glob Public Health. 2025 Jan 24;5(1):e0004165. doi: 10.1371/journal.pgph.0004165 (PMC11760041; doi:10.1371/journal.pgph.0004165)
Supplement: Attachment — Submitted filename: PGPH-D-24-01383_ReviewerComments_2024-08-08.docx [file pgph.0004165.s001.docx]

1. Abstract: Reconsider use of “infectious” when discussing complications. “Infectious” implies these complications are communicable, as opposed to being “related to infection”, which I believe the authors intend.
2. Introduction:
   a. 1^st^ paragraph: Suggest listing possible serious complications of varicella, as this is an important topic covered in the rest of the manuscript.
3. Materials and Methods:
   a. 1^st^ paragraph: What proportion of the population of Guatemala and Guatemala City are covered by these four hospitals?
   b. 1^st^ paragraph: (2^nd^ sentence) Change “where” to “who were”.
   c. 2^nd^ paragraph: Where was the assumption of 2.5 workdays lost implemented in your analysis? If this was part of the calculation for indirect costs in Table 4, there should be a footnote in the table indicating that 2.5 workdays lost was an assumption, not a value derived from patient data.
   d. 3^rd^ paragraph: (2^nd^ & 3^rd^ sentences) Is the “average exchange rate” averaged over the entire period from 2015 to 2019? Or were quetzales converted to USD for each year using *that year’s* average exchange rate? What is the currency year in which results are presented?
   e. 3^rd^ paragraph: (4^th^ & 5^th^ sentences) This information belongs in the results section.
4. Results:
   a. 2^nd^ paragraph (below Table 1): Again, reconsider the use of the word “infectious”.
   b. 5^th^ paragraph: Explain what kind of “image” was utilized in HCRU.
   c. 6^th^ paragraph: What is meant by “human resources”? Does this mean the type of specialist employed?
   d. Table 2: Are these values *per patient*? For example, on average did each patient have 10.7 laboratory tests during their course of illness with varicella?
   e. Table 2: Please capitalize “Surgeries”.
   f. Table 3: As for Table 2, are these values *per patient*? For example, for a patient with complicated varicella, did this patient have 14 consultations with an infectologist? Or is it that, out of 124 varicella cases tracked from 2015-2019, there were 14 visits with infectologists, some of which may have been repeat visits for the same patient? This needs to be explained more clearly in the text and the table caption.
   g. Table 3: (for General Ward stay) Change “días” to “days”.
   h. 7^th^ paragraph: (below Table 3, 2^nd^ sentence) Is this the **average** total cost of care? Also, please add decimals to “USD 2,892 (SD 4,179)” to reflect the same level of confidence as for the remaining values in that same sentence.
   i. Table 4: (Indirect costs) The heading of “Caregiver days lost from work” implies that the values shown are the number of workdays lost, i.e., that for an uncomplicated case of varicella the caregiver lost roughly 3 months of work. Perhaps change heading to “Cost of caregiver days lost from work” to add clarity.
   j. 9^th^ paragraph: (2^nd^ paragraph below Table 4, 2^nd^ sentence) It seems counterintuitive that complications would be less frequent in immunocompromised patients and those treated at UNOP. Perhaps add a sentence explaining this to the 2^nd^ paragraph of the Discussion, which comments on variation of treatment protocols between institutions.
5. Discussion:
   a. 3^rd^ paragraph: (1^st^ sentence) “In this study, the average hospital stay was longer…” Longer than what?
   b. 4^th^ paragraph: (3^rd^ sentence) Please provide the values for average duration of hospital stay, % of patients requiring ICU, and duration of ICU stay, for comparison purposes with the values presented here for other countries.
   c. 6^th^ paragraph: (1^st^ sentence) Add “in Guatemala” to the end of the sentence to clarify the point.
   d. 9^th^ paragraph – and throughout the Discussion in general: Please reword sentences so it is clear when you are discussion Guatemala results and when you are discussing other countries. In this paragraph, in the 4^th^ sentence, are these results for Guatemala or Mexico? This is very confusing throughout the Discussion section.
   e. 10^th^ paragraph: (3^rd^ sentence: “… which have the highest costs”) The highest costs out of what group? European countries? Countries included in reference #10?
   f. 11^th^ paragraph: (1^st^ sentence) Add “in patients in Guatemala” to the end of the sentence to clarify which country you are discussing.
   g. 13^th^ paragraph: (1^st^ sentence: “There is no country-level data on the incidence of varicella and its complications…”) Is this for Guatemala specifically? Or for all of LATAM?
   h. 13^th^ paragraph: (2^nd^ sentence) What is the reference for these figures? Are the citations at the end of this paragraph meant to apply to only the final sentence of the paragraph, or throughout? Appropriate citations should be listed with the sentence in which the specific data is presented to avoid confusion.
   i. 14^th^ paragraph: Same comment as above regarding citations.
   j. 13^th^ – 16^th^ paragraphs: These paragraphs would fit better in the Introduction.
