# Supplementary material for: Clinical and economic burden of varicella in pediatric patients hospitalized in four institutions in Guatemala
Source: PLOS Glob Public Health. 2025 Jan 24;5(1):e0004165. doi: 10.1371/journal.pgph.0004165 (PMC11760041; doi:10.1371/journal.pgph.0004165)
Supplement: Attachment — Submitted filename: Response to Reviewers.docx [file pgph.0004165.s002.docx]

1. Abstract: Reconsider use of “infectious” when discussing complications. “Infectious” implies these complications are communicable, as opposed to being “related to infection”, which I believe the authors intend.

R/ Wording was changed.

2. Introduction:
   a. 1^st^ paragraph: Suggest listing possible serious complications of varicella, as this is an important topic covered in the rest of the manuscript.

R/ The following text was added: Although it generally has a benign course, it can also result in serious complications like bacterial superinfection of skin lesion with or without bacterial sepsis, central nervous system involvement, pneumonia, other complications are myocarditis, corneal lesions, nephritis, arthritis, acute glomerulonephritis, hepatitis, and thrombocytopenia

3. Materials and Methods:
   a. 1^st^ paragraph: What proportion of the population of Guatemala and Guatemala City are covered by these four hospitals?

R/	There is no official proportion of the population that these hospitals cover. However, as they are the largest reference hospitals in the country, they attend to most of the complicated varicella cases from the metropolitan area and some from the rest of the country.

b. 1^st^ paragraph: (2^nd^ sentence) Change “where” to “who were”.

R/	the change was made

c. 2^nd^ paragraph: Where was the assumption of 2.5 workdays lost implemented in your analysis? If this was part of the calculation for indirect costs in Table 4, there should be a footnote in the table indicating that 2.5 workdays lost was an assumption, not a value derived from patient data.

R/ It was an assumption for most caregivers since it was not recorded in the medical record. The next footnote was added: The assumption of 2.5 workdays lost was implemented in the analysis as part of the calculation for indirect costs, it was an assumption based on previously published literature, not a value derived from patient-specific data.

d. 3^rd^ paragraph: (2^nd^ & 3^rd^ sentences) Is the “average exchange rate” averaged over the entire period from 2015 to 2019? Or were quetzales converted to USD for each year using *that year’s* average exchange rate? What is the currency year in which results are presented?

R/ Since the costs were obtained from 2015 to 2019, they were converted from quetzales using the average exchange rate for that period and adjusted for inflation to reflect 2022 values. Additionally, some costs from earlier years were also adjusted for inflation.

We changed the wording in the paragraph for more clarity: Direct costs were calculated using data from national forms, insurance, and official national sources. All costs were expressed in local currency (quetzales), and an average exchange rate from the national bank for the entire period from 2015 to 2019 was applied (1 USD = 7.579926 quetzales). Costs prior to the study period were adjusted for inflation. For the year 2022, the reported exchange rate was 7.76808 quetzales per 1 USD. Additionally, all costs were adjusted for cumulative inflation up to 2022 to reflect their updated value.

e. 3^rd^ paragraph: (4^th^ & 5^th^ sentences) This information belongs in the results section.

R/ It was change in results, 5^th^ paragraph, 2^nd^ and 3^rd^ sentences. We left in the same location the 6^th^ sentence “To calculate the cost of a daily workday, data on the national average minimum wage of USD 11.91 (Q. 93.55) in 2022 were used”

4. Results:
   a. 2^nd^ paragraph (below Table 1): Again, reconsider the use of the word “infectious”.

R/ was changed to “Related to infection”

b. 5^th^ paragraph: Explain what kind of “image” was utilized in HCRU.

R/ The next was add: image per patient were utilized, the most common was the X-rays

c. 6^th^ paragraph: What is meant by “human resources”? Does this mean the type of specialist employed?

R/ Yes, human resources is the type of specialist employed

d. Table 2: Are these values *per patient*? For example, on average did each patient have 10.7 laboratory tests during their course of illness with varicella?

R/Yes, the name of the table was change for clarity to “Average use of health resources per patient associated with varicella in four institutions in Guatemala City, 2015 to 2019”

e. Table 2: Please capitalize “Surgeries”.

R/ It was fixed

f. Table 3: As for Table 2, are these values *per patient*? For example, for a patient with complicated varicella, did this patient have 14 consultations with an infectologist? Or is it that, out of 124 varicella cases tracked from 2015-2019, there were 14 visits with infectologists, some of which may have been repeat visits for the same patient? This needs to be explained more clearly in the text and the table caption.

R/ Repeat visits to the same specialist were not included in the 124 patients. 14 patients required evaluation by an infectious disease specialist. Wording was changed for clarity to “Table 3 describes the HCRU of the total 124 patients, including the types of specialists involved and the number of evaluations by other specialists. The total number of visits from one specialist was not taken into account.”

g. Table 3: (for General Ward stay) Change “días” to “days”.

R/ It was fixed

h. 7^th^ paragraph: (below Table 3, 2^nd^ sentence) Is this the **average** total cost of care? Also, please add decimals to “USD 2,892 (SD 4,179)” to reflect the same level of confidence as for the remaining values in that same sentence.

R/ Yes, this is the average total cost of care, we add: “was USD 2891.55 (SD 4179.35)”

i. Table 4: (Indirect costs) The heading of “Caregiver days lost from work” implies that the values shown are the number of workdays lost, i.e., that for an uncomplicated case of varicella the caregiver lost roughly 3 months of work. Perhaps change heading to “Cost of caregiver days lost from work” to add clarity.

R/ it was change to “Cost of caregiver days lost from work”

j. 9^th^ paragraph: (2^nd^ paragraph below Table 4, 2^nd^ sentence) It seems counterintuitive that complications would be less frequent in immunocompromised patients and those treated at UNOP. Perhaps add a sentence explaining this to the 2^nd^ paragraph of the Discussion, which comments on variation of treatment protocols between institutions.

R/ The next was add in the 3^rd^ sentence: In this institution, patients are admitted even when they do not present complications, whereas other hospitals only admit patients with infectious and other complications

5. Discussion:
   a. 3^rd^ paragraph: (1^st^ sentence) “In this study, the average hospital stay was longer…” Longer than what?

R/ It was clarified “In this study, the average hospital stay was longer compared with data from the study conducted by Wolfson et al. (2018), with a notable percentage of patients requiring intensive care and a prolonged ICU stay.”

b. 4^th^ paragraph: (3^rd^ sentence) Please provide the values for average duration of hospital stay, % of patients requiring ICU, and duration of ICU stay, for comparison purposes with the values presented here for other countries.

R/ The next was clarified “The length of hospital stay could be longer due to complications, but compared to reports from Peru and Argentina, complication rates were lower. Another reason for the prolonged hospital stay could be the profile of the patients, as a large proportion had comorbidities, especially cancer. These data indicate that, although the percentage of patients required ICU admission is lower, the average hospital stay in pediatric intensive care **(7.5 days for 21.8% of patients)** is longer compared to countries such as Argentina, which has an average of less than 4.8 days, and Peru, with an average of 7.0 days”

c. 6^th^ paragraph: (1^st^ sentence) Add “in Guatemala” to the end of the sentence to clarify the point.

R/ “in Guatemala” was added.

d. 9^th^ paragraph – and throughout the Discussion in general: Please reword sentences so it is clear when you are discussion Guatemala results and when you are discussing other countries. In this paragraph, in the 4^th^ sentence, are these results for Guatemala or Mexico? This is very confusing throughout the Discussion section.

R/We reworded sentences, hope will be easier to follow:

“For prescribed medications in Guatemala, patients used an average of 1.6 over-the-counter medications and received 2.3 antibiotics. Additionally, 84.2% of patients were taking some medication, with an average of 2.1 medications per patient. This is similar to what was reported in Poland, where 1.5 antibiotic prescriptions per patient were recorded. However, the average number of antibiotics per patient in Guatemala is slightly lower than the 2.5 antibiotics per patient used in Mexico. In comparison, 94% of patients in Peru and Poland were reported to be taking at least one medication, while in Argentina this figure was 77.3%”

e. 10^th^ paragraph: (3^rd^ sentence: “… which have the highest costs”) The highest costs out of what group? European countries? Countries included in reference #10?

R/Yes, in the European countries included in reference #10 it was change to 11, this is what we added for clarification “Regarding costs, the expense per hospitalized patient is Guatemala is lower than in Mexico, where the cost is 5,786.20 USD per patient. However, Guatemala’s cost is higher compared to other Latin American countries, such as Argentina (2,947.70 USD) and Peru (769.90 USD) per hospitalized patient. These costs are significantly lower than the health resource utilization (HRU) observed in European countries such as Hungary and Poland, as reported in the study by Wolfson et al. (2019), which identified these countries as having the highest costs. These costs will be important to consider when evaluating the potential inclusion of the varicella vaccine in the national immunization program”

f. 11^th^ paragraph: (1^st^ sentence) Add “in patients in Guatemala” to the end of the sentence to clarify which country you are discussing.

R/ The sentence was fixed “In comparison with other Latin American literature, the complication rate was lower in patients in Guatemala”

g. 13^th^ paragraph: (1^st^ sentence: “There is no country-level data on the incidence of varicella and its complications…”) Is this for Guatemala specifically? Or for all of LATAM?

R/ This is for Guatemala, the sentence was fixed “There is no country-level data on the incidence of varicella and its complications for Guatemala”

h. 13^th^ paragraph: (2^nd^ sentence) What is the reference for these figures? Are the citations at the end of this paragraph meant to apply to only the final sentence of the paragraph, or throughout? Appropriate citations should be listed with the sentence in which the specific data is presented to avoid confusion.

R/We add de citations at the end of the sentence for clarification:

“However, according to conservative estimates, varicella is responsible for 4.2 million serious complications leading to hospitalization and 4,200 deaths worldwide each year **(14)**. In the pre-vaccination era, in high-income developed countries, the case fatality rate for varicella was 3 deaths per 100,000 cases **(17).** In Costa Rica, during this period, more than 80% of cases occurred in children under 14 years of age, resulting in the hospitalization of 872 children over a 10-year period, of which 631 (72.4%) were under 12 years of age **(18)**. Annual hospitalization rates and similar fatalities are estimated in cases of natural varicella as in outbreaks, with hospitalization rates of 0.297%, 0.129%, 0.147%, 0.269% for children ages under 1, 1 to 4, 5 to 9, and 10 to 14 years, respectively; and mortality rates of 0.0106%, 0.0027%, 0.0018%, 0.0031% for children under 1, 1 to 4, 5 to 9, and 10 to 14 years, respectively **(19)**. Using these data to estimate cases in Guatemala, and considering the INE (National Institute of Statistics Guatemala) population, it is estimated that by 2024 there will be a population of 4,809,312 children under 12 years of age. Extrapolating the data annually for the Guatemalan population, an estimated 30,000 cases (28,800-38,400) of patients with varicella are projected. A total of hospitalized children is expected by age, being 396, 567, 664, and 1,030 for the groups under 1, 1 to 4, 5 to 9, and 10 to 14 years, respectively, for a total of 2,657 children. According to studies in Brazil, 12-month-old children have a higher risk of complications (14.3%), while for other ages a lower percentage (5.7%) of complications is recorded **(14)**. Therefore, 57 children under one year of age and 148 children between 1 and 14 years of age are expected to present complications, for an estimated total of 205 affected. Annual costs are estimated to be USD 777,614.20 (SD 1,000,031), with a prognosis of 46 deaths.”

i. 14^th^ paragraph: Same comment as above regarding citations.

R/ We add de citations at the end of the sentence for clarification:

The effectiveness of universal vaccination against varicella has been widely studied in various countries, demonstrating striking reductions in the incidence of complications and hospitalizations. For example, in Germany, after the introduction of routine vaccination, a 90% reduction in complications was observed in children under 5 years of age, which represented a significant milestone in the protection of children's health **(20).** In Costa Rica, where the vaccine was implemented in 2007, an impressive 95% coverage was achieved by 2015, resulting in a 73.8% decrease in varicella cases and a massive 98% reduction in hospital complications **(18)** . Argentina, after the introduction of the vaccine in 2015, with coverage of 77.6% by 2019, also experienced a notable decrease of 83.9% in the incidence of the disease **(21)**. In Colombia, introduced into the vaccination scheme in 2015 with 90% coverage, a 70.5% reduction in incidence and a significant decrease in general mortality was evident **(15)**. Furthermore, experiences such as that of Uruguay, where a complication rate of 7% was found in vaccinated children compared to 12% in unvaccinated children **(14)**, underline the positive impact of vaccination in protecting children's health throughout the region. These findings support universal vaccination as an effective strategy to reduce both the burden of disease and the costs associated with healthcare in Latin America

j. 13^th^ – 16^th^ paragraphs: These paragraphs would fit better in the Introduction.

R/ After careful consideration, we, the authors, prefer to keep these paragraphs in their current placement

**Additionally, we received another review via email**

3. Have the authors made all data underlying the findings in their manuscript fully available (please refer to the Data Availability Statement at the start of the manuscript PDF file)?

R/ We publish the data at the following link: <https://figshare.com/s/239f6aa09df2135de997> for private link, we are planning to do a public, in csv format.

5. Review Comments to the Author

Please use the space provided to explain your answers to the questions above. You may also include additional comments for the author, including concerns about dual publication, research ethics, or publication ethics. (Please upload your review as an attachment if it exceeds 20,000 characters)

Reviewer #1: Although the authors have described this as a retrospective descriptive study, it would be useful to use some statistical parameter to confirm the difference between the group of patients with complications versus patients without complications, e.g. Chi-square test.

in the page 10, show: "while factors such as age, sex, ethnicity, immunocompromise, and outcome did not show clinically significant differences (p < 0.05)" Should be expressed as p>0.05.

R/ The change was madre in the 8^th^ paragraph of results, was add the paragraph 9 and 10^th^ (Now 7 page.

We change the next: Comparing the demographic and epidemiological characteristics of patients with and without complications, it was observed that the frequency of complications varied depending on the hospital (p < 0.001), while factors such as age, sex, ethnicity, immunocompromise, and outcome did not show clinically significant differences (p > 0.05).

To the next paragraph: In comparing patients with varicella who developed complications to those who did not, significant associations were observed with hospital location and immunocompromised status. Patients treated at certain hospitals exhibited higher rates of complications (χ² = 18.23, p < 0.001), suggesting variability in outcomes across facilities. Immunocompromised patients, particularly those with cancer, were significantly more likely to experience complications (χ² = 18.36, p < 0.001). No significant differences were found between the groups regarding age, gender, ethnicity, area of residence, or nutritional status, indicating these factors did not significantly influence the likelihood of complications in this cohort

When comparing patients with varicella who developed complications to those who did not, significant differences were observed in healthcare utilization patterns. Patients with complications were more likely to consult a specialist, with 62.3% doing so compared to 31.0% of non-complicated cases (χ² = 11.64, p < 0.001). They also had a higher number of specialist consultations, with 24.4% of complicated cases receiving four or more consultations, whereas none were reported in the non-complicated group (χ² = 13.70, p < 0.001). Additionally, patients with complications had more outpatient visits, with 41.5% having at least one visit compared to 19.0% of non-complicated patients (χ² = 6.31, p = 0.012), indicating a greater demand for medical attention among those who developed complications.

Independent samples Student's t-tests were conducted to compare the length of hospital stay between varicella patients who developed complications and those who did not. Patients with complications had a significantly longer general ward stay (Mean = 9 days, SD = 10) compared to non-complicated patients (Mean = 5 days, SD = 3), t(106) = 3.34, p = 0.001. Similarly, the total bedridden stay was longer for complicated cases (Mean = 11 days, SD = 12) versus non-complicated cases (Mean = 5 days, SD = 3), t(99) = 4.28, p < 0.001. A t-test could not be performed for ICU length of stay because the non-complicated group showed no variation (Mean = 0 days, SD = 0). These results indicate that developing complications are associated with a longer hospital stay in varicella patients.

Reviewer #2: Please see my attached review.
R/ It was reviewed.

The only additional comments I have, other than outlined in the attachment, are that (1) dates when the site was accessed should be included in references for websites, and (2) if possible, a URL (and access date) should be provided for reference #4.

R/ The URL was provided but the reference #4 was change to reference #5. “Sistema Nacional de Vigilancia Epidemiológica -SINAVE. Protocolo del Sistema Nacional de Vigilancia Epidemiológica SINAVE [Internet]. Guatemala: Ministerio de Salud Pública y Asistencia Social; 2018 [cited 2022 Jan 2]. Available from: [**https://pesquisa.bvsalud.org/portal/resource/pt/biblio-1025059**](https://pesquisa.bvsalud.org/portal/resource/pt/biblio-1025059)”

We also add the date accessed in reference #8 , #10

**Journal Requirements:**

1.  Please provide additional details regarding participant consent. If you are reporting a retrospective study of medical records or archived samples, please ensure that you have discussed whether the IRB or ethics committee waived the requirement for informed consent. If patients provided informed written consent to have data from their medical records used in research, please include this information.

R/ The next was add for clarification. “We confirm that the study was reviewed by the Independent Ethics Committee and the respective research committees, which determined that informed consent was not required for this retrospective study of medical records.”

For clarification to the editorial, When the study was submitted to the various committees, informed consent was not included in the submission requirements. Each committee reviewed the study and confirmed that it was not necessary.

2. In the online submission form, you indicated that "The data that support the findings of this study are available on request from the corresponding author".

All PLOS journals now require all data underlying the findings described in their manuscript to be freely available to other researchers, either 
a. In a public repository, 
b. Within the manuscript itself, or 
c. Uploaded as supplementary information.

R/ We publish the data at the following link: <https://figshare.com/s/239f6aa09df2135de997> for private link, we are planning to do a public, in csv format.
