# Supplementary material for: Clinical and economic burden of varicella in pediatric patients hospitalized in four institutions in Guatemala
Source: PLOS Glob Public Health. 2025 Jan 24;5(1):e0004165. doi: 10.1371/journal.pgph.0004165 (PMC11760041; doi:10.1371/journal.pgph.0004165)
Supplement: Attachment — Submitted filename: Response_to_Reviewers_auresp_2.docx [file pgph.0004165.s003.docx]

**Comments to the Author**

1. If the authors have adequately addressed your comments raised in a previous round of review and you feel that this manuscript is now acceptable for publication, you may indicate that here to bypass the “Comments to the Author” section, enter your conflict of interest statement in the “Confidential to Editor” section, and submit your "Accept" recommendation.

Reviewer #1: (No Response)

Reviewer #2: (No Response)

2. Does this manuscript meet PLOS Global Public Health’s [publication criteria](https://track.editorialmanager.com/CL0/https:%2F%2Fjournals.plos.org%2Fglobalpublichealth%2Fs%2Fcriteria-for-publication/1/010f0193cc71d5fd-734115b9-d7ef-47c4-99b2-51a996409bd0-000000/9oNQbiCLcPIbv7EY3AftZtZ1wSI6scfIc1_K94CoamY=189)? Is the manuscript technically sound, and do the data support the conclusions? The manuscript must describe methodologically and ethically rigorous research with conclusions that are appropriately drawn based on the data presented.

Reviewer #1: Yes

Reviewer #2: Yes

3. Has the statistical analysis been performed appropriately and rigorously?

Reviewer #1: Yes

Reviewer #2: I don't know

R/ Could you kindly specify the areas of concern so that we can address them and provide further clarification?

4. Have the authors made all data underlying the findings in their manuscript fully available (please refer to the Data Availability Statement at the start of the manuscript PDF file)?

The [PLOS Data policy](https://track.editorialmanager.com/CL0/https:%2F%2Fjournals.plos.org%2Fglobalpublichealth%2Fs%2Fdata-availability/1/010f0193cc71d5fd-734115b9-d7ef-47c4-99b2-51a996409bd0-000000/DYgl8w0ENYzlBLXyRCuVfn9Zvkx7etNB4HKLpD7SgzA=189) requires authors to make all data underlying the findings described in their manuscript fully available without restriction, with rare exception. The data should be provided as part of the manuscript or its supporting information, or deposited to a public repository. For example, in addition to summary statistics, the data points behind means, medians and variance measures should be available. If there are restrictions on publicly sharing data—e.g. participant privacy or use of data from a third party—those must be specified.

Reviewer #1: No

Reviewer #2: Yes

We added a folder to the Figshare repository containing the database in CSV format, along with a text file describing the variables for better understanding. However, the DOI has changed and can now be accessed at <https://doi.org/10.6084/m9.figshare.28057484>.

5. Is the manuscript presented in an intelligible fashion and written in standard English?

PLOS Global Public Health does not copyedit accepted manuscripts, so the language in submitted articles must be clear, correct, and unambiguous. Any typographical or grammatical errors should be corrected at revision, so please note any specific errors here.

Reviewer #1: Yes

Reviewer #2: Yes

6. Review Comments to the Author

Please use the space provided to explain your answers to the questions above. You may also include additional comments for the author, including concerns about dual publication, research ethics, or publication ethics. (Please upload your review as an attachment if it exceeds 20,000 characters)

Reviewer #1: Citation number 4 is still missing the correct reference.
As well as quotes 8 and 9. The access day is missing.

R/ We add to clarification ”Giglio et al. (2018) reported that up to 28% of outpatients and 98.7% of hospitalized patients experienced one or more complications (4).”

Reviewer #2: This updated version of the manuscript is much tighter and cleaner than the previous version -- well done! I have only a few minor comments remaining:
1. Materials and Methods, 1st paragraph, last sentence -- "... and where hospitalized" -- Please change "where" to "who were".

R/ The change was made
2. Results, 3rd paragraph below Table 4, 3rd sentence -- "They also had a higher number of pa specialist consutations..." -- what is "pa"?

R/ It was deleted because it was a typo.

3. Overall -- Please double check all citation numbers. The citation number for Giglio in the final paragraph of the Discussion is incorrect; I have not confirmed the rest of the citations.

R/ The double check was completed, and citation number 22 was removed because it corresponded to a previous discussion that had already been deleted. The correct number for Giglio was made.

In Paragraph 4, O'Connor's bibliography was removed as it did not contribute any relevant information to that specific paragraph. It has been relocated to Discussion, Paragraph 11, in the 10 sentences: “O’Connor et al. (2014) reported a mortality rate of 12% due to viral infections, including varicella, in pediatric patients with acute lymphoblastic leukemia (15).” O’Connor is now listed as Reference 15.
